# The past, present and future distribution of a deep-sea shrimp in the Southern Ocean

**DOI:** 10.7717/peerj.1713

**Published:** 2016-02-23

**Authors:** Zeenatul Basher, Mark J. Costello

**Affiliations:** Institute of Marine Science, Leigh Marine Laboratory, The University of Auckland, Auckland, New Zealand

**Keywords:** Climate change, Last glacial maximum, Benthos, Range shift, Biogeography, Refugia, Antarctica, Decapod, Species distribution modeling

## Abstract

Shrimps have a widespread distribution across the shelf, slope and seamount regions of the Southern Ocean. Studies of Antarctic organisms have shown that individual species and higher taxa display different degrees of sensitivity and adaptability in response to environmental change. We use species distribution models to predict changes in the geographic range of the deep-sea Antarctic shrimp *Nematocarcinus lanceopes* under changing climatic conditions from the Last Glacial Maximum to the present and to the year 2100. The present distribution range indicates a pole-ward shift of the shrimp population since the last glaciation. This occurred by colonization of slopes from nearby refugia located around the northern part of Scotia Arc, southern tip of South America, South Georgia, Bouvet Island, southern tip of the Campbell plateau and Kerguelen plateau. By 2100, the shrimp are likely to expand their distribution in east Antarctica but have a continued pole-ward contraction in west Antarctica. The range extension and contraction process followed by the deep-sea shrimp provide a geographic context of how other deep-sea Antarctic species may have survived during the last glaciation and may endure with projected changing climatic conditions in the future.

## Introduction

The response of organisms to a changing environment depends on their capacity to cope with the physiological cost imposed by the new conditions and dispersal capacities (Peck, 2004; Peck, 2005; Ingels et al., 2012). Species commonly react to climate change by shifting their latitudinal range (Perry et al., 2005; Parmesan, 2006; Dulvy et al., 2008; Hiddink & Ter Hofstede, 2008; Cheung et al., 2012; Cheung, Watson & Pauly, 2013). Many organisms living in the Antarctic have evolved to survive the combined physiological and ecological constraints of the cold environment (Thatje et al., 2008). During the last glacial maximum (LGM, ca. 19.5–16 ka; Gersonde et al., 2005), Antarctic marine life was challenged by even more extreme environmental conditions with reduced shallow habitat area on the continental shelf and a scarcity of food in the open ocean (i.e., primary production is higher close to coast than open ocean) (Smith & Comiso, 2008). This forced them to take refuge in ice free regions, and then re-colonize their present range (Aronson et al., 2007; Barnes & Conlan, 2007; Thatje et al., 2008). At present, Antarctic ecosystems are experiencing significant environmental changes with the retreat of glaciers and the disintegration of ice shelves due to climate warming suggesting a southward shift of pelagic and benthic communities in the future (Turner et al., 2009). Average global temperature is expected to increase approximately 2 °C in the next 100 years (IPCC Climate Change, 2007). Although, satellite data indicate sea ice extent has not changed markedly over the last 25 years (Bjørgo, Johannessen & Miles, 1997), recent studies suggested ice cover is changing due to climate warming, generally decreasing but increasing in some regions (Polvani & Smith, 2013; Rignot et al., 2013; Simmonds, 2015). The Intergovernmental Panel for Climate Change predicts that a net loss of 25% sea ice cove over the next 100 years would result in a reduced extent of phytoplankton productivity around the Southern Ocean (SO). This may alter food webs through reduced food access coupled with higher metabolic demands due to the warming climate.

The first phylogeographic study of Antarctic shrimps suggested that there was a postglacial expansion of the shelf-inhabiting species *Chorismus antarcticus,* but not of the deep-water shrimp *Nematocarcinus lanceopes* (Raupach et al., 2010). Benthic shelf species have been more affected by glaciations than pelagic or deep sea inhabiting species (Janko et al., 2007). However, deep-sea ecosystems may experience abrupt environment changes, such as variation in particulate organic carbon, changing current oscillation pattern etc. (Smith & Kaufmann, 1999; Ruhl & Smith, 2004; Smith et al., 2013). Tropical deep-sea ecosystems fauna may be vulnerable to relatively small changes in temperature (Danovaro, Dell’Anno & Pusceddu, 2004) and so may cold stenothermal polar species (Barnes, Griffiths & Kaiser, 2009). The re-colonization of areas in the Antarctic deep-sea by predators (e.g., litholids) due to climate warming was shown in several past studies (Thatje et al., 2005a; Aronson et al., 2007; Aronson et al., 2009; Griffiths et al., 2013; Kaiser et al., 2013).

Past study methods for single species range-shifts range from spatially explicit mechanistic models (Hill et al., 2001) to climate driven bioclimatic envelope based (Walther, Berger & Sykes, 2005) and correlative species distribution models (SDM) (Peterson & Vieglais, 2001; Pearson et al., 2002; Pearson & Dawson, 2003; Graham et al., 2004; Thuiller et al., 2005; Waltari et al., 2007; Peterson et al., 2011; Bentlage et al., 2013). SDM can provide insights into potential climate warming effects on species even when their physiological limitations are poorly known (Crumpacker, Box & Hardin, 2001; Elith, Kearney & Phillips, 2010). Dambach et al. (2012) used SDM to predict how Antarctic shrimp ranges contracted during the LGM, but did not predict future ranges. In order to understand how shrimps could have survived through past climatic events and how they could respond to future climate change, we ran a SDM using a more comprehensive set of distribution records of the shrimp *Nematocarcinus lanceopes* and environmental variables representing Past, Present and Future climate conditions. *Nematocarcinus lanceopes* was selected because it had the most extended distribution records of a deep-sea benthic (Kirkwood, 1984; Arntz et al., 2006; Basher & Costello, 2014). Our findings show how other deep-sea Antarctic species may have survived during the last glaciation and may endure with projected changing climatic conditions in the future.

## Materials & Methods

### Study area and observation data

**Figure 1:**
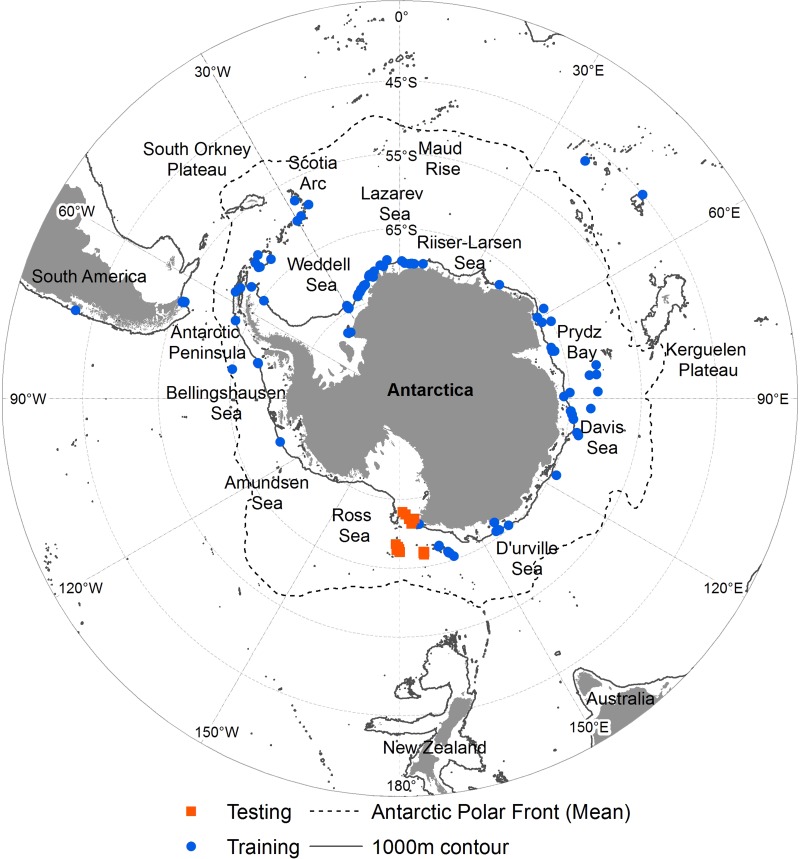
Occurrence of *N. lanceopes* in the Southern Ocean. ‘Circles’ represent the locations used for model training and ‘squares’ represent the locations used for independent model testing.

Our study area lies in the Southern Ocean between north of the Antarctic Circumpolar Current (ACC) close to 40 °N and the Antarctic coast in the south (Fig. 1). The bathymetry is dominated by deep ocean ridges and a continental shelf break at ca 1,000 m, which is two to four times deeper than the shelf break in other oceanic regions (Knox, 2006). A strong temperature gradient of 4 °C over 0.5° of latitude across the Subtropical front (Sikes et al., 2009) and the ACC distinguishes the Southern Ocean from northern temperate waters. The ACC is the strongest current on Earth and connects the Atlantic, Pacific and Indian ocean basins (Rintoul, Hughes & Olbers, 2001). The ACC creates a physical barrier that has isolated Antarctica for 25 million years (Clarke, Barnes & Hodgson, 2005).

A total of 87 *N. lanceopes* observation records were obtained from the Ocean Biogeographic Information System (OBIS, 2011), the SCAR-Marine Biodiversity Information Network (De Broyer & Danis, 2011), and literature (Fig. 1 and Table S1). An additional 30 records from a recent cruise in the Ross Sea were used for model validation (Basher, Bowden & Costello, 2014a). All records were filtered to remove apparent geographic errors (i.e., coordinates plotting on land or in different regions) before combining them into a single dataset for model training or validation using ArcGIS (ESRI, 2011). All of the data used have been submitted to SCAR MARBIN for open-access online publication following publication of Basher & Costello (2014). The data will thus also become accessible through OBIS and GBIF.

### Environmental data

Environmental data were obtained from the Global Marine Environment Datasets (GMED) (Basher, Costello & Bowden, 2014b), namely depth, temperature, salinity, ice cover and primary productivity. The variables were derived from remotely sensed and *in-situ* measured datasets, and had a spatial resolution (pixel size) of 5 arc-min or ca. 9 km near the equator. As shrimps are predominantly benthic, we used environmental variables reflecting environment conditions near seabed (e.g., in Present and Future models). Unfortunately no seabed environmental layers were available for paleo (i.e., Past) conditions, thus we selected surface layers as a proxy of the seabed conditions. The data set for the past (i.e., LGM) comprised of depth (Depth, m), ice thickness (IceT, m), surface salinity (sSal, ppt) and sea surface temperature (SST, °C). Bottom temperature and salinity layers were only available for Present and Future layers. The dataset for Present and Future conditions was comprised of depth (Depth, m), sea bottom salinity (bSal, ppt), sea bottom temperature (SBT, °C), ice cover (IceC, 0–1%) and primary productivity (Prod, mgC m^−2^/day).We used the Institut Pierre Simon Laplace (IPSL; http://icmc.ipsl.fr/) Future climate A2 scenario for the environmental data of the year 2100. Our scenario selection was limited to A2 as the deep-sea data layers in other climate scenarios were not available and generating them for this specific study by compiling raw data was beyond the scope of the study. The Depth in Future scenario was considered the same as Present depth since Future predictions of sea level change were currently not available. All variables were derived from mean annual average of *in-situ* or satellite data . (see Basher, Costello & Bowden, 2014b for details about all layers). High correlations between environmental predictors may not only show spurious results as well as negatively affect SDM performance and its transferability through space and time (Heikkinen et al., 2006; Bigg et al., 2008; Jiménez-Valverde et al., 2009; Liu, White & Newell, 2009; Dormann et al., 2013). None of the environmental variables used in our models showed strong correlations (*R*^2^ > 0.7) when tested for pair-wise correlations using Pearson’s correlation.

### Model building

MaxEnt 3.3.3e (Phillips, Anderson & Schapire, 2006) was used to model the current distribution of *N. lanceopes* and to project Past and Future distribution ranges. The program uses a machine learning algorithm following the principles of maximum entropy (Jaynes, 1982). Reviews comparing up to 16 models and of >200 taxa found that machine-learning methods including MaxEnt consistently outperformed traditional linear methods (Elith et al., 2006; Meißner et al., 2014) and that presence-only models were preferable because limited sampling can increase the prevalence of false absences within a dataset. MaxEnt starts with a uniform distribution during the modelling process, and successively fits the model to the data (occurrence records and environmental variables). MaxEnt repeatedly tests the predictive capability of the model and improves by iteratively permuting and varying the input variables and features thereof. This is measured in the log likelihood or “model gain”, which illustrates the discrepancy between the model identified distribution and the uniform distribution (Elith et al., 2011). MaxEnt thus specifies the relative suitability of the environment (interpreted as the potential geographic distribution) of the study organism.

MaxEnt models were generated using 10 bootstrap replicate runs with 100,000 random background points. The average of the 10 predictions across all replicates was used for further analysis. We excluded duplicate records that fell within individual pixels of background environment layers on each dataset using ‘Remove duplicate presence records’ feature in the MaxEnt software. The occurrence records were also split into 75% for training and 25% for testing for bootstrap replications. We set the maximum iterations to 1,000, to facilitate model convergence. As suggested by Phillips & Dudik (2008) the default regularization (i.e., smoothing) value was used because it results in better performance of evaluation data for presence-only datasets. We minimized unreliable extrapolation into areas with environmental conditions that were not encountered during model training using the ‘fade by clamping’ option of the software. Any predicted areas having the prediction value below the Minimum Presence Threshold (MPT) were considered unsuitable for the species. Models were projected onto ‘Past’ and ‘Future’ environmental datasets at the end of the iteration phase in two separate instances. As the final procedure, in ArcGIS 10 (ESRI, 2011) we calculated the species range shift maps by subtracting Past SDM raster from the Present SDM raster and then the Present SDM raster from the Future raster to get the Present and Future change maps respectively.

### Model evaluation

The logistic model output format gives a predicted suitability value ranging from 0 (unsuitable) to 1 (optimal) (Phillips & Dudik, 2008). The final output raster was classified into four classes based on the range of predicted suitability value: HS (High Suitability, 0.75-Maximum); MS (Medium Suitability, 0.5–0.75); LS (Low Suitability, MPT-0.5) and NS (Not suitable, Values below MPT). These classified raster files were used to interpret the suitability of *N. lanceopes* environment in the Southern Ocean. MaxEnt allows for model evaluation by the Area Under the Receiver Operating Characteristic Curve (AUC) (Phillips, Dudík & Schapire, 2004). AUC is a threshold-independent measurement of model discrimination. An AUC value of 0.5 indicates model predictions are not better than random and AUC > 0.9 indicates high performance (Peterson et al., 2011). We used a random data split approach to evaluate model performance using a bootstrap procedure with an evaluation dataset (25% of the entire Present species distribution records). We used percent variable contribution and jack-knife procedures in the software to investigate the relative importance of different environmental predictors. The jack-knife procedure produces a model by using variables in isolation to examine how well the result fits the known model gain (for both training and test data). Response curves were used to evaluate the relationships between environmental variables and the predicted presence probability of *N. lanceopes*. Confidence maps were generated using the ratio of the standard deviation of the MaxEnt prediction maps to the mean environment suitability. Using an independent dataset is the optimal method for evaluating model performance (Phillips & Dudik, 2008; Kumar & Stohlgren, 2009). Probability of occurrence values, which ranged from 0 to 1, where 0 meant no probability of presence and 1 meant highest probability of presence at that particular location, were extracted from the average of all bootstrap models on each data set using the “Extract Values to Point” function of Spatial Analyst in ArcGIS. We evaluated model accuracy with the independent dataset by seeing how successfully the model predicted the species’ potential distribution outside its given sampling locations.

## Results

### Predicted distributions

**Figure 2:**
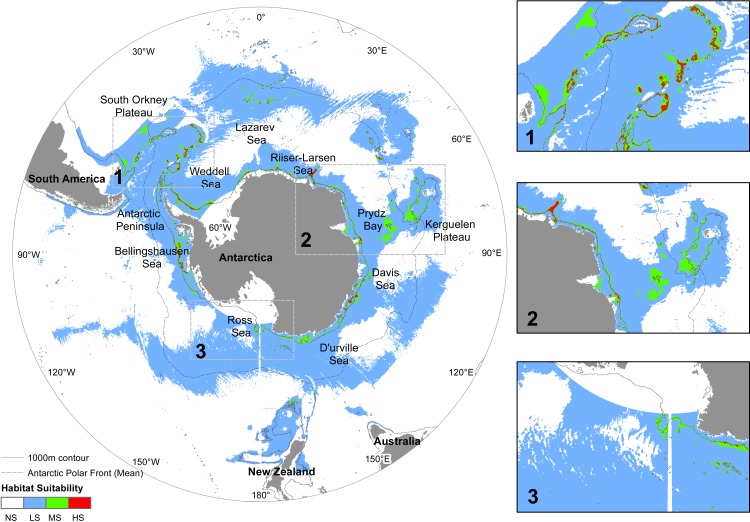
Predicted distribution of *N. lanceopes* during the Last Glacial Maximum. Environment suitability: HS, High suitability (red); MS, Medium suitability (green); LS, Low suitability (sky); NS, Not suitable (white). Close up maps of: 1, Scotia Arc and Antarctic Peninsula; 2, Prydz Bay and Kerguelen Plateau; 3, Ross Sea and Amundsen Sea showed in the close up boxes on the right.

All the SDM had a high predictive power based on the values of AUC > 0.95 (AUC ± SD, Past 0.950 ± 0.01; Future 0.968 ± 0.008). The minimum presence threshold (MPT) values were 0.012 and 0.015 for Past and Future models respectively. The relative importance of the environmental variables to the SDM showed that depth had the highest explanatory power 61–79% for both Past (Table 1 and Fig. 2) and Future (Table 1 and Fig. 4) climate conditions. The second and third most important variables were temperature (26% for Past) and ice cover (9% for Future) (Table 1) (Fig. S2). Independent records used to validate model were all plotted into areas having prediction value above MPT suggests high predictive performance of all the models.

**Table 1:** Summary of MaxEnt results from the Past and Future models. The high values of ‘Contribution’ and ‘Permutation Importance’ indicated that Depth, Temperature and Ice Cover were the main predictors regulating the distribution of *N. lanceopes* in the Southern Ocean. ‘Without predictor’ values indicated model performance when models were developed with all other variables excluding that individual predictor and ‘Only with predictor’ indicated models developed with only that predictor.

Model Summary	Past				Future				
Training samples	54				54				
Test samples	18				18				
Training gain	2.17				2.51				
Training AUC ± SD	0.950 ± 0.01				0.968 ± 0.008				
Test AUC ± SD	0.903 ± 0.03				0.956 ± 0.02				
Minimum presence threshold	0.012				0.015				
**Predictors influence**									
	Depth	SST	sSal	IceT	Depth	SBT	bSal	Prod	IceC
Contribution (%)	79.57	18.42	1.02	0.99	61.03	5.27	0.07	2.51	31.12
Permutation importance	71.91	26.43	1.49	0.16	88.29	0.74	0.02	2.1	8.84
**Without predictor**									
Training gain	0.53	1.89	2.15	2.15	1.28	2.44	2.51	2.43	2.22
Test gain	−0.56	0.68	0.4	1.74	1.43	2.53	2.63	2.6	2.28
AUC	0.699	0.91	0.904	0.922	0.902	0.954	0.956	0.962	0.944
**Only with predictor**									
Training Gain	1.75	0.47	0.14	0.04	1.76	0.92	0	0.13	0.76
Test gain	1.77	−0.02	0.13	−0.03	1.78	1.06	0	0.09	0.86
AUC	0.92	0.704	0.63	0.493	0.928	0.866	0.528	0.658	0.845

**Figure 3:**
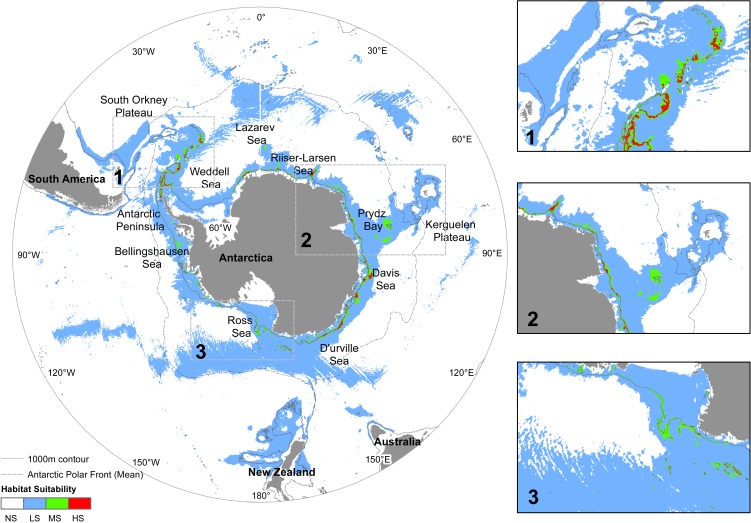
Predicted distribution of *N. lanceopes* at Present. Environment suitability: HS, High suitability (red); MS, Medium suitability (green); LS, Low suitability (sky); NS, Not suitable (white). Close up maps of: 1, Scotia Arc and Antarctic Peninsula; 2, Prydz Bay and Kerguelen Plateau; 3, Ross Sea and Amundsen Sea showed in the close up boxes on the right.

### Past (LGM) distribution

The predicted distribution for the Past indicated that *N. lanceopes* would have been widely distributed in the Sub-Antarctic regions near the Scotia Arc (South Georgia, South Orkney, South Sandwich Islands), Kerguelen Plateau, Mawson Sea, D’Urville Sea and in the Bellingshausen Sea (Fig. 2). The maximum predicted environment suitability value was 0.875 (Table 1). The high confidence in prediction indicated optimum model performance in identifying potential glacial refugia (i.e., areas with persistent population over time) (Fig. S4). Model predicted about 30 million-km^2^ area (i.e., sum of LS, MS and HS) suitable for *N. lanceopes* environment during LGM. More than half of the areas (62%) were identified as ‘not suitable’ for *N. lanceopes*. The areas having low, medium and high environment suitability were 36%, 2% and 0.5% respectively (Fig. 5).

**Figure 4:**
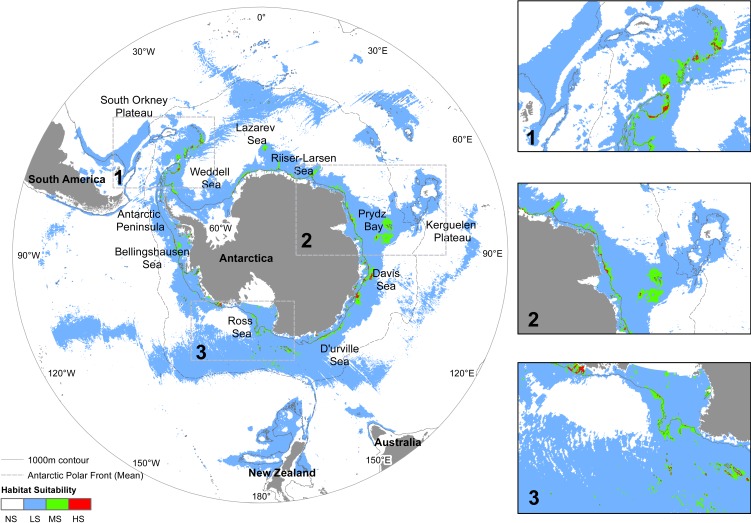
Predicted distribution of *N. lanceopes* in the Future (year 2100). Environment suitability: HS, High suitability (red); MS, Medium suitability (green); LS, Low suitability (sky); NS, Not suitable (white). Close up maps of: 1, Scotia Arc and Antarctic Peninsula; 2, Prydz Bay and Kerguelen Plateau; 3, Ross Sea and Amundsen Sea showed in the close up boxes on the right.

**Figure 5:**
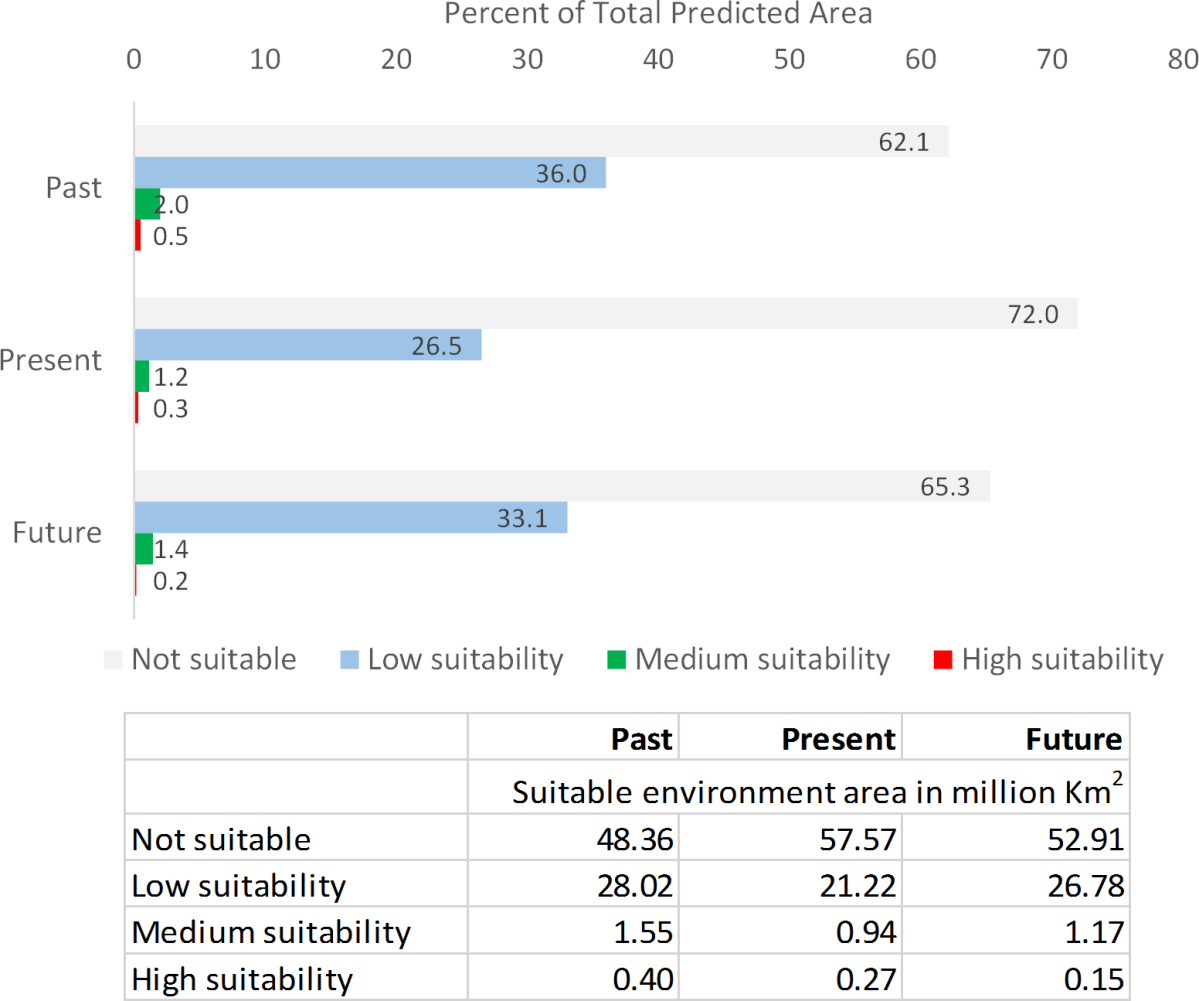
Variation in the area identified as suitable environment for *N. lanceopes* in the MaxEnt model predictions. Environment suitability in the graph: Not suitable (grey); Low suitability (sky); Medium suitability (green); High suitability (red).

### Present distribution

The predicted Present distribution covered the current known distribution range of the species. The highest predicted suitability was in areas near the Mawson Sea, Kerguelen Plateau, Ross Sea slope, Davos Sea, Prydz Bay, South Orkney Islands, Bellingshausen Sea and at Gunnerus Ridge in between Riiser-Larsen and the Cosmonaut Sea (Fig. 3). The Present distribution range suggested a pole-ward shift of *N. lanceopes* after the LGM by colonization of previously unoccupied slope areas. All of the independent validation records occurred in areas having medium to high probability of predicted *N. lanceopes* distribution (Fig. 1). The model predicted a contraction of suitable environment in the present since the LGM day (22.5 million km^2^), because more areas (72%) identified as ‘not suitable’ environments in compared to the Past (Fig. 5).

### Future distribution

The SDM under the predicted Future (2100) climate conditions showed further contraction of *N. lanceopes* distribution, although there was an increase in suitable areas in the deeper slope regions (Figs. 4 and 7). The potential range predicted by the model showed range expansion into the deeper sections of the eastern Ross Sea shelf, areas between Amundsen Sea and Ross Sea, slopes of D’Urville Sea, Prydz Bay, Maud Rise, bathyal (i.e., seabed) regions of Mawson Sea, Prydz Bay and to the Aurora Canyon near the eastern tip of the Antarctic Peninsula (Fig. 4). The maximum predicted environment suitability value was 0.94 (Table 1). However, the predicted areas with ‘high suitability’ values continued to decrease (0.18%) in the Future, while and and environment areas with ‘low suitability’ and ‘medium suitability’ increased slightly (33%) and (1.45%) respectively (Fig. 5). The model predicted an overall expanded distribution (28 million km^2^) in Future, with all of the potential expansion areas adjacent to existing *N. lanceopes* populations. Thus, these areas would be likely to be colonised (Figs. 4 and 7).

**Figure 6:**
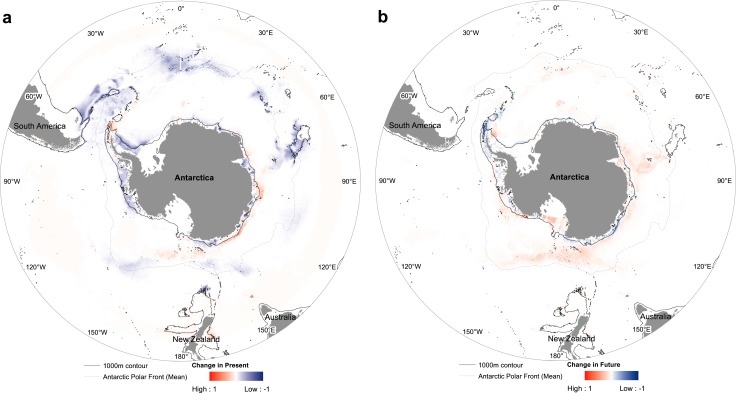
*Nematocarcinus lanceopes* range loss and gain from (A) Past last glacial maximum period to Present day, (B) from Present to Future. Areas in ‘red’ indicate gained range and areas in ‘blue’ lost range.

### Effect of climate change

The results indicated a pole-ward shift between the predicted distribution of Past (LGM) and Present day, and Present to Future (year 2100). The highly suitable LGM areas located in the Sub-Antarctic (South Sandwich, South Orkney Islands, and South Georgia), Bouvet Island, Western Weddell Sea and the Kerguelen plateau regions became contracted into smaller areas now. The model also suggested colonization of slope areas of east Antarctica (D’Urville Sea, Davis Sea, and Ross Sea) and the tip of the Antarctic Peninsula (Fig. 6A).

**Figure 7:**
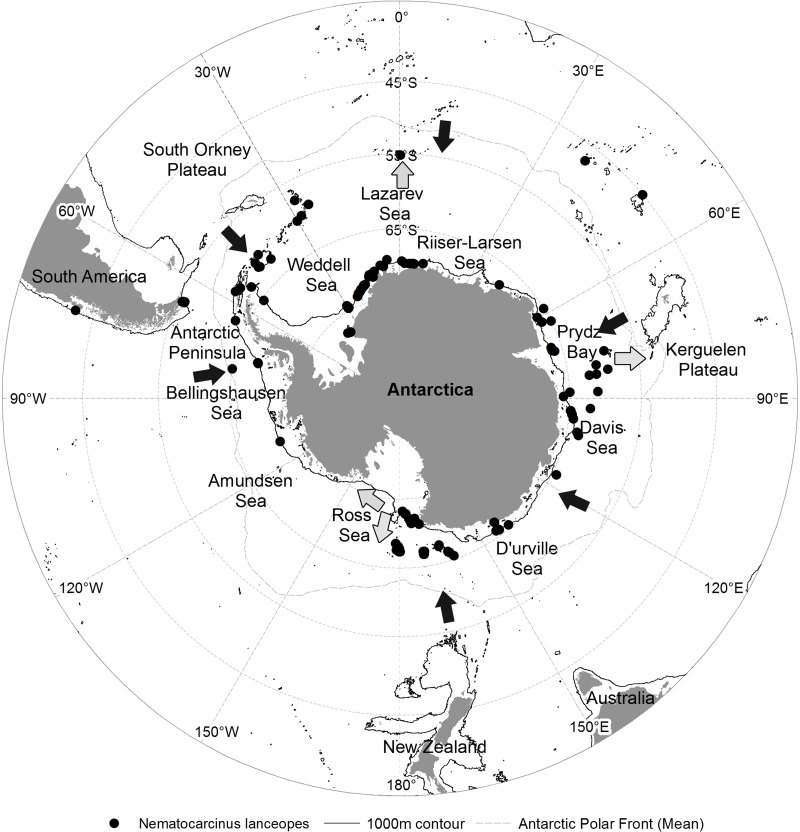
Predicted range contraction and expansion direction of *N. lanceopes* populations in the Southern Ocean based on the model predictions of Past, Present and Future environment conditions in relation to present population locations. Contraction (black arrow), expansion (grey arrow) and present population locations (black dots).

The predicted distribution for the Future followed the previous trend of pole-ward range shift of *N. lanceopes* populations. However, a range expansion was predicted into newer regions of deeper slope areas near the Scott Seamount in the Ross Sea, Marie Byrd Seamounts in the Amundsen Sea, Aurora Canyon in the eastern tip of the Antarctic Peninsula, and Maud Rise north of the Lazarev Sea (Fig. 6B). Nevertheless, the predicted change in area was not the same for all Antarctic regions. It contracted more in the western Antarctic (Antarctic Peninsula) and expanded more in the Eastern Antarctic regions, i.e. Bellingshausen Sea and eastern Ross Sea. The eastern Ross Sea, which currently covered by ice all year round, was predicted to have more open ocean (i.e., ice free) areas in the Future (Future ice cover in Figs. S1 and S3C) which would facilitate *N. lanceopes* colonization from the nearby slope areas in the west (Fig. 7).

## Discussion

*Nematocarcinus lanceopes* is the most widely distributed deep-sea shrimp in the Southern Ocean (Arntz & Gorny, 1991; Gutt, Gorny & Arntz, 1991; Arntz et al., 1999; Guzmán & Quiroga, 2005; Lovrich et al., 2005; Thatje, Bacardit & Arntz, 2005b; Donnelly, Sutton & Torres, 2006; Basher & Costello, 2014). We found that the geographic distribution of *N. lanceopes* was most influenced by depth, ice cover and temperature; supporting previous studies (Dambach et al., 2012; Basher, Bowden & Costello, 2014a). As found by Barnes, Griffiths & Kaiser (2009) for Antarctic benthic gastropods and bivalves, *N. lanceopes* in our models showed a contraction and expansion of distribution following the variation of ice cover (Fig. 7). This suggests that our findings are more widely applicable to benthic species in Antarctica.

### Temporal prediction of species range extension

Species distribution models can predict the direction of species range contractions or expansions (Araújo et al., 2005) but projections beyond the temporal range of a training dataset (i.e., distribution in future dates) require a cautious interpretation to avoid potential pitfalls. The AUC value tends to increase when the selected background area is larger than the species observed presence area (Phillips & Dudik, 2008; Merow, Smith & Silander, 2013). Although using AUC as the only method of model validation has its own caveats (Jiménez-Valverde & Lobo, 2007; Lobo, Jimenez-Valverde & Real, 2008; Pineda & Lobo, 2009), it has been used widely in SDM studies for past and future climate conditions (Lobo, Jimenez-Valverde & Hortal, 2010; Varela, Lobo & Hortal, 2011; Dambach et al., 2012; Weinmann et al., 2013). In addition to AUC, we used model confidence maps and found a consistency in predictive power of the models to characterize the distribution of the species in different temporal resolution, and identified regions that contained less variation in predictions (Fig. S4). All of the three confidence maps have high confidence values for our predictions; indicating that all of the modelled predictions were likely to reflect actual distributions range for the species (Phillips, Anderson & Schapire, 2006; Anderson & Gonzalez Jr, 2011; Davies & Guinotte, 2011).

Many shelf and slope inhabiting Antarctic fauna have an extended bathymetric range (Brey et al., 1996; Basher & Costello, 2014) akin to deep-sea organisms in other oceans (Clarke, 2003). This suggests that Antarctic fauna may represent an evolutionary history of movement in and out of deep water, driven by glacial cycles (Aronson et al., 2007; Fraser et al., 2012). During the LGM, turbidity and currents due to ice scour were likely to have affected the survival of fauna on the continental slope around Antarctica (Thatje, Hillenbrand & Larter, 2005c; Thatje et al., 2008). For most benthic taxa, survival was possible in deep-sea refugia close to the Antarctic continent due to open ocean polynya that supplied food from primary production at the surface (Thatje et al., 2008). Our Past model also suggested LGM refugia around the northern part of the Scotia Arc, southern tip of South America, South Georgia, Bouvet Island, southern tip of the Campbell Plateau and Kerguelen Plateau (Fig. 2). The refugia near Campbell Plateau and Bouvet Island were not identified in a previous study by Dambach et al. (2012) due to their more limited data. Molecular studies on Antarctic isopods, amphipods, and bivalves have indicated similar re-colonization events in nearby shelf and slope from glacial refugia in several taxa since the LGM (Rogers, 2007; Newman et al., 2009). The shrimp refugia found in this study complement these molecular studies and provide a geographic context of how species ranges adjust to environmental change by moving up and down the continental slope and on and off the continental shelf (Fig. 7).

The Antarctic Peninsula warmed 3.7 ± 1.6 °C over the last century (Vaughan et al., 2003; Clarke et al., 2007), while areas in Halley and Amundsen-Scott at the South Pole cooled (Turner et al., 2005). Sea ice cover in the Amundsen Sea reduced over the last three decades and the trend seems set to continue in Future (Fig. S1 and Rignot et al., 2013). Food availability in the deep sea is dependent upon the surface productivity and vertical supply of organic matter from the upper ocean (Smith & Comiso, 2008). Thus, an increase in food availability in the deep sea generally triggers a significant meiofaunal response (Gooday, 2002) resulting in an increase of overall benthic biomass (Levin et al., 2001). As sea ice melts, new environment areas will become available in the shelf and slope for re-colonization which will be supported with increased projected chlorophyll-a production areas (Shepherd, Wingham & Rignot, 2004; Whitehouse et al., 2008; Gerringa et al., 2012).

The overall suitable environmental areas for benthic shrimps in the Antarctic Peninsula shrinks in the Present and Future models compared to the Past model (Figs. 2, 3 and 7). In contrast, the Amundsen Sea has increased suitable area from the Past to the Future models (Figs. 3, 4 and 7). Other regions where environment suitability is projected to increase in the Future include the deeper slopes of the Kerguelen Plateau and the eastern Ross Sea. The Kerguelen Plateau is one of the major linear shelves near Antarctica and has a strong temperature gradient compared to the Antarctic Peninsula and Victoria Land areas. This makes this area likely to experience thermally driven range shifts of Antarctic fauna (Barnes, Griffiths & Kaiser, 2009). With projected warming of the temperature and decreased ice coverage around these regions in the next 100 years, *N. lanceopes* is likely to expand in these regions (Fig. 4).

## Conclusion

We modelled the potential distribution of the deep-sea shrimp *Nematocarcinus lanceopes* in the Southern Ocean. Results indicated a contraction of suitable environment from the Sub-Antarctic regions and pole-ward expansion on the continental slopes from the LGM to Present, and Present to year 2100. However, an expansion of areas with a suitable environment in the future was predicted for eastern Antarctica but contraction in the western Antarctic. Further research should examine how typical these changes will be of other Southern Ocean species and how benthic communities and food webs will change.

## Supplemental Information

10.7717/peerj.1713/supp-1 Table S1 Locations and source of *Nematocarcinus lanceopes* records used for the model training and validation

10.7717/peerj.1713/supp-2 Figure S1 Environmental layers used for modelling in this study Scales range from high (red), to low (blue).

10.7717/peerj.1713/supp-3 Figure S2 Influence of environmental variables in the model prediction performance

10.7717/peerj.1713/supp-4 Figure S3 Unclassified MaxEnt prediction maps of (A) past, (B) present and (C) future distribution of *N. lanceopes*

10.7717/peerj.1713/supp-5 Figure S4 Prediction confidence maps of past, present and future MaxEnt models of *N. lanceopes* (from left to right) Black indicates high confidence or less variation in predicted performance among all replicates.

## Additional Information and Declarations

### Data Availability

The following information was supplied regarding data availability: The research in this article did not generate any raw data and all datasets used for the research are publicly available from different sources which were mentioned in Supplemental Information.
