# Supplementary material for: The past, present and future distribution of a deep-sea shrimp in the Southern Ocean
Source: PeerJ. 2016 Feb 23;4:e1713. doi: 10.7717/peerj.1713 (PMC4768674; doi:10.7717/peerj.1713)
Supplement: Table S1 [file peerj-04-1713-s001.docx]

**The past, present and future distribution of a deep-sea shrimp in the Southern Ocean**

**Zeenatul Basher^*^ and Mark J. Costello**

Institute of Marine Science, The University of Auckland, Auckland 1142, New Zealand.

**Supplementary Information**

Table S1. Locations and source of *Nematocarcinus lanceopes* records used for the model training and validation.

| Type/Collection | Institute Code | Locations | Source |
| --- | --- | --- | --- |
| Database |  | 6 | De Broyer C and Danis B (Editors). SCAR-MarBIN: The Antarctic Marine Biodiversity Information Network. 01-Aug-2013. World Wide Web electronic publication. Available online at http://www.scarmarbin.be/ |
|  | AAD | 18 | De Broyer C and Danis B (Editors). SCAR-MarBIN: The Antarctic Marine Biodiversity Information Network. 01-Aug-2013. World Wide Web electronic publication. Available online at http://www.scarmarbin.be/ |
|  | AADC | 10 | De Broyer C and Danis B (Editors). SCAR-MarBIN: The Antarctic Marine Biodiversity Information Network. 01-Aug-2013. World Wide Web electronic publication. Available online at http://www.scarmarbin.be/ |
|  | AWI | 6 | De Broyer C and Danis B (Editors). SCAR-MarBIN: The Antarctic Marine Biodiversity Information Network. 01-Aug-2013. World Wide Web electronic publication. Available online at http://www.scarmarbin.be/ |
|  | BAS | 2 | De Broyer C and Danis B (Editors). SCAR-MarBIN: The Antarctic Marine Biodiversity Information Network. 01-Aug-2013. World Wide Web electronic publication. Available online at http://www.scarmarbin.be/ |
|  | SAMC | 2 | De Broyer C and Danis B (Editors). SCAR-MarBIN: The Antarctic Marine Biodiversity Information Network. 01-Aug-2013. World Wide Web electronic publication. Available online at http://www.scarmarbin.be/ |
|  | SMF | 1 | De Broyer C and Danis B (Editors). SCAR-MarBIN: The Antarctic Marine Biodiversity Information Network. 01-Aug-2013. World Wide Web electronic publication. Available online at http://www.scarmarbin.be/ |
| Journal Article | - | 32 | Dambach, J., Thatje, S., Rödder, D., Basher, Z., Raupach, M.J. 2012. Effects of Late-Cenozoic glaciation on habitat availability in Antarctic benthic shrimps (Crustacea: Decapoda: Caridea). PLoS ONE, 7(9), e46283. doi:10.1371/journal.pone.0046283. |
|  | AWI | 44 | Gorny, M. 1999. On the biogeography and ecology of the Southern Ocean decapod fauna. Scientia Marina 63 (Supl. 1): 367-382. |
|  | UoI | 1 | Guzmán, G., Quiroga, E. 2005. New records of shrimps (Decapoda: Caridea and Dendrobranchiata) in deep waters of Chile. Gayana (Concepcin), 69(2), 285-290 |
|  | CBM | 1 | Komai T. & Segonzac M. 2005. — Two new species of Nematocarcinus A. Milne-Edwards,1881 (Crustacea, Decapoda, Caridea, Nematocarcinidae) from hydrothermal vents on the North and South East Pacific Rise. Zoosystema 27 (2): 343-364. |
|  | BAS | 6 | Linse, K., Griffiths, H.J., Barnes, D.K.A., Brandt, A., Davey, N., David, B., De Grave, S., D′Udekem D′Acoz, C., Eléaume, M., Glover, A.G., Hemery, L.G., Mah, C., Martín-Ledo, R., Munilla, T., O′Loughlin, M., Pierrat, B., Saucède, T., Sands, C.J., Strugnell, J.M., Enderlein, P. 2013. The macro- and megabenthic fauna on the continental shelf of the eastern Amundsen Sea, Antarctica. Continental Shelf Research, 68(0), 80–90. doi:10.1016/j.csr.2013.08.012. |
|  | AWI | 1 | Thatje, S., Bacardit, R., & Arntz, W. (2005). Larvae of the deep-sea nematocarcinidae (Crustacea : Decapoda : Caridea) from the southern ocean. Polar Biology, 28(4), 290-302. DOI 10.1007/s00300-004-0687-0 |
|  | MNHN | 3 | Basher, Z., & Costello, M. J. (2014). Crustacea: Decapoda: shrimps. In K. P. De Broyer C., Griffiths H.J., Raymond B., Udekem d’Acoz C. d’, Van de Putte A.P., Danis B., David B., Grant S., Gutt J., Held C., Hosie G., Huettmann F., Post A., Ropert-Coudert Y. (Ed.), Biogeographic Atlas of the Southern Ocean (pp. 190-194). Cambridge: Scientific Committee on Antarctic Research |
|  | NIWA | 550 | Basher, Z., Bowden, D. A., & Costello, M. J. (2014). Diversity and distribution of deep-sea shrimps in the Ross Sea region of Antarctica. PLoS ONE, 9(7), e103195. |
| Museum | USNM | 1 | Department of Invertebrate Zoology, Research and Collections Information System, NMNH, Smithsonian Institution. See: http://www.mnh.si.edu/rc/db/collection_db_policy1.html, 05-14-2010 |
| Expedition Report | NIWA | 8 | NIWA OBIS; Available at nzobis.niwa.co.nz |

* Records used for independent model validation.
