# Supplementary figures and images for: The past, present and future distribution of a deep-sea shrimp in the Southern Ocean
Source: PeerJ. 2016 Feb 23;4:e1713. doi: 10.7717/peerj.1713 (PMC4768674; doi:10.7717/peerj.1713)

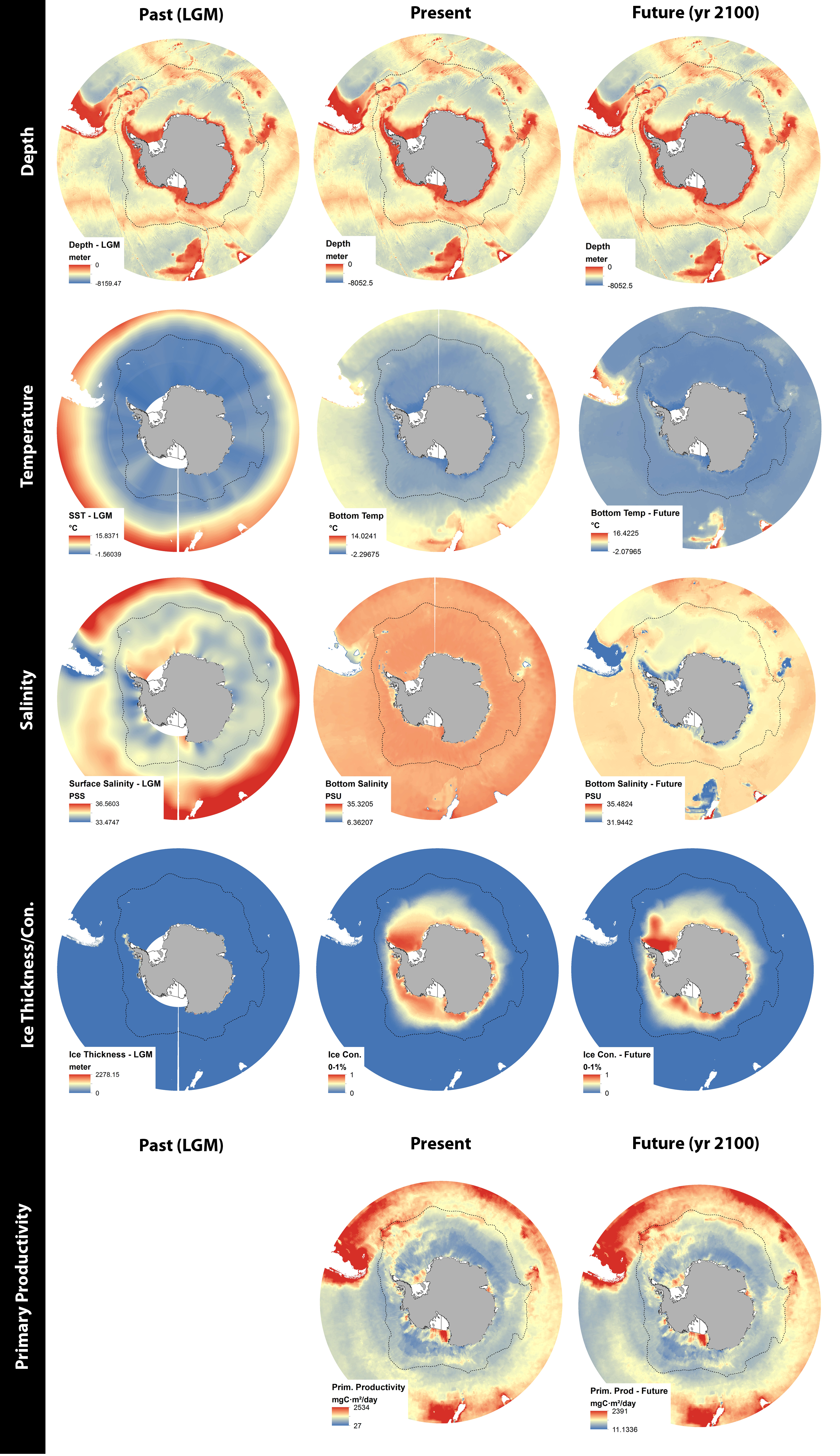

Supplement: Figure S1 — Scales range from high (red), to low (blue). [file peerj-04-1713-s002.jpg]

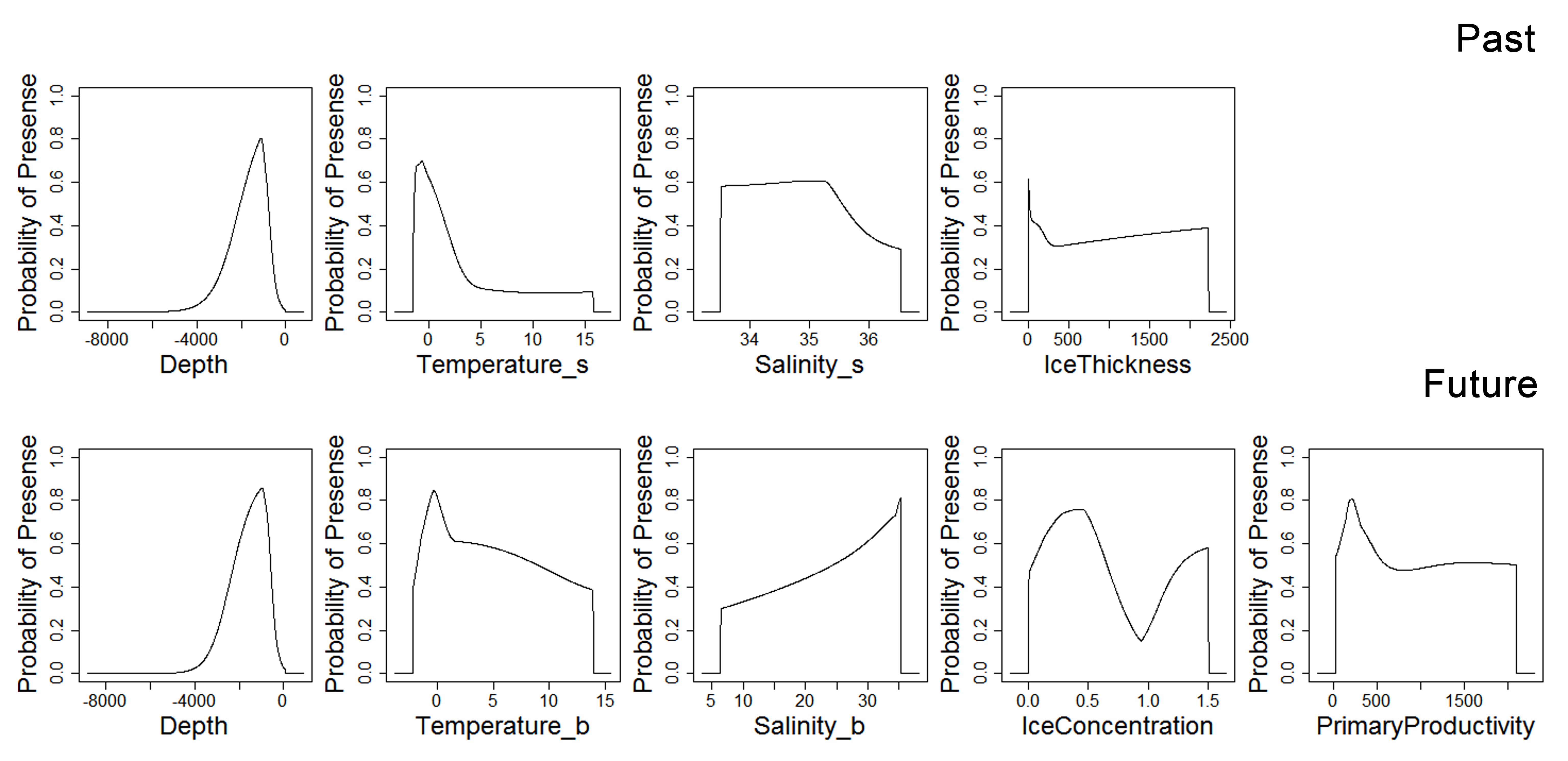

Supplement: Figure S2 [file peerj-04-1713-s003.jpg]

A

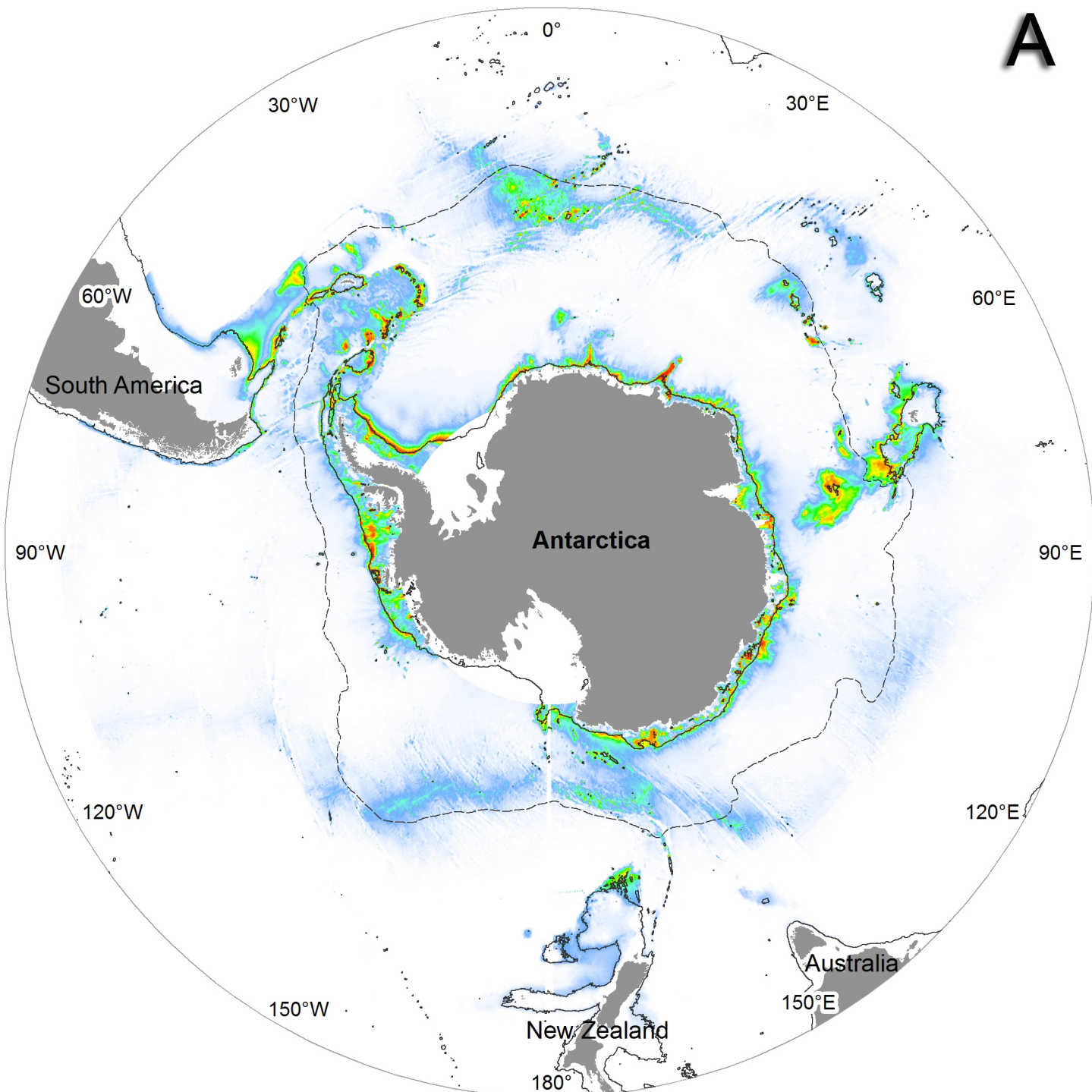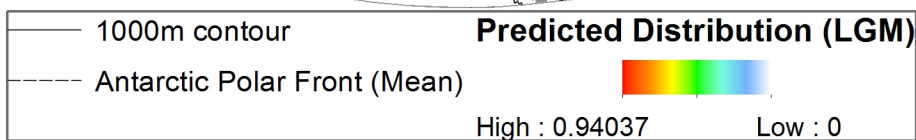

**B**

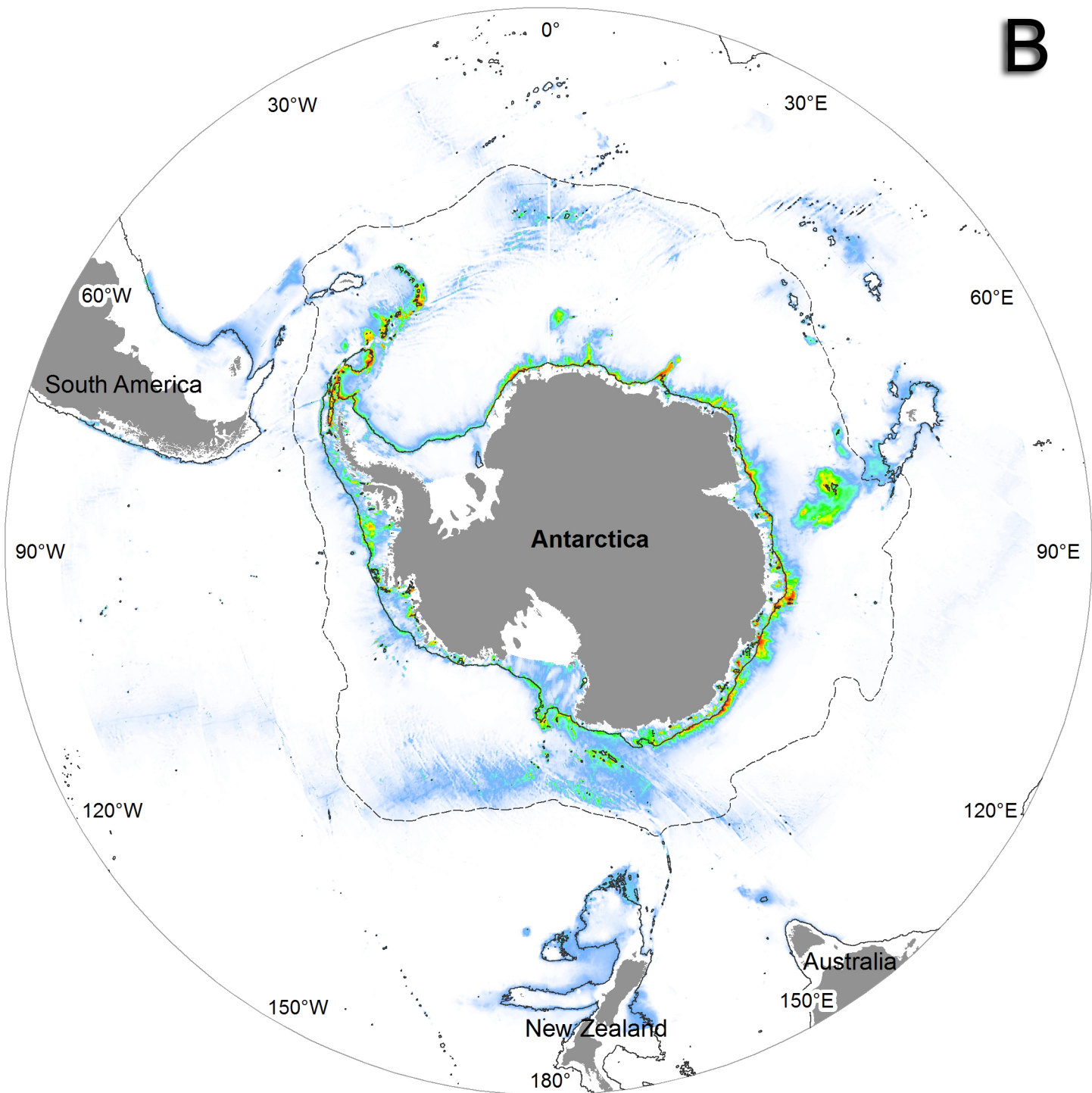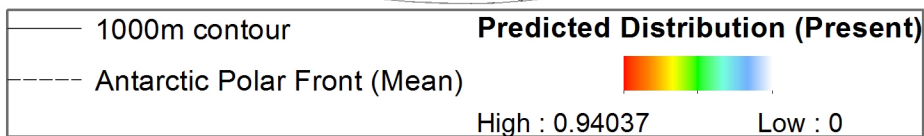

C

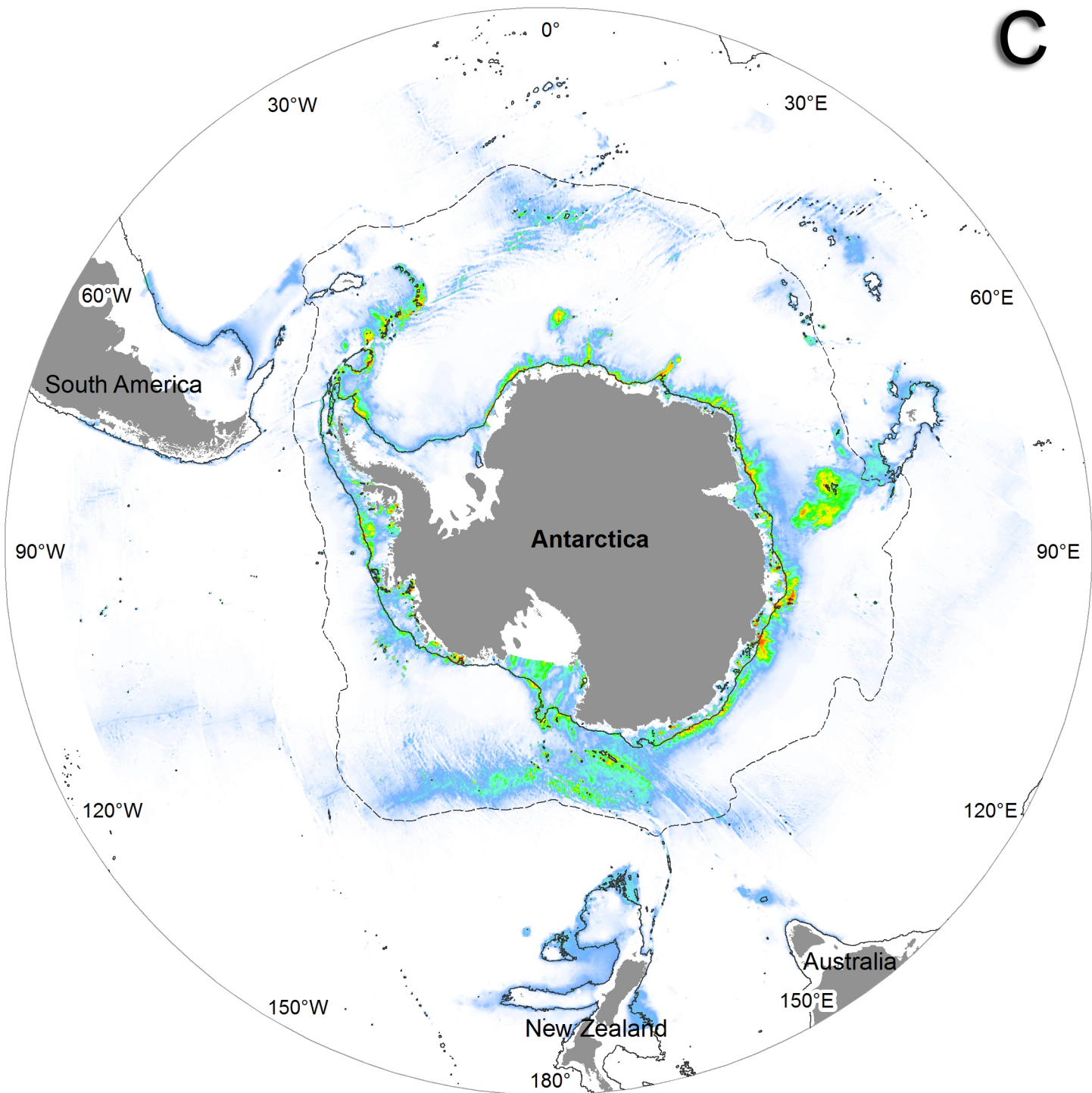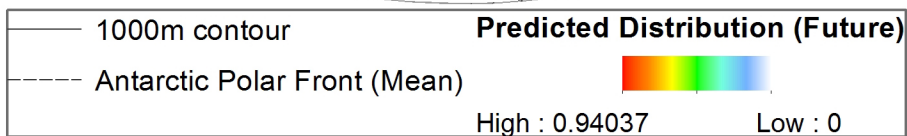

Supplement: Figure S3 [file peerj-04-1713-s004.pdf]

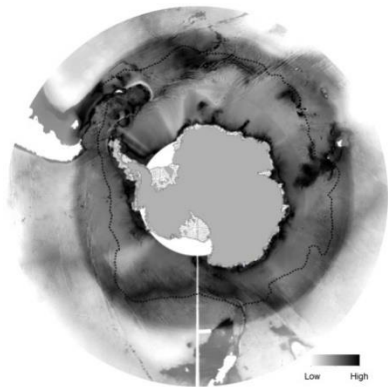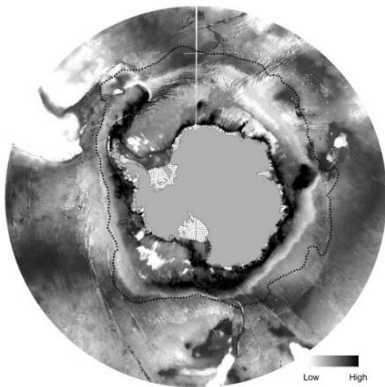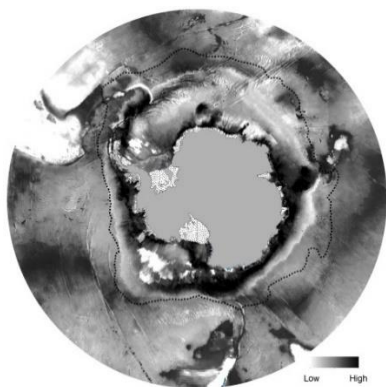

Supplement: Figure S4 — Black indicates high confidence or less variation in predicted performance among all replicates. [file peerj-04-1713-s005.pdf]
